# Pearls and Pitfalls of Revision Unilateral Biportal Endoscopic Lumbar Spine Surgery: A Technical Note

**DOI:** 10.7759/cureus.86958

**Published:** 2025-06-29

**Authors:** Jiawen Fong, Zi Xian Justin Chou, Walter-Soon-Yaw Wong, Yilun Huang

**Affiliations:** 1 Orthopaedic Surgery, Sengkang General Hospital, Singapore, SGP; 2 Orthopaedic Surgery, Total Orthopaedic Care and Surgery, Singapore, SGP

**Keywords:** decompression, endoscopic spine surgery, endoscopic technique, minimally invasive, revision surgery

## Abstract

We aim to describe our revision unilateral biportal endoscopic lumbar spine surgery (RUBELSS) approach, emphasising pre-operative planning, surgical technique, and management of complications. Key steps include utilising various endoscopic approaches based on the compression site, pre-operative magnetic resonance imaging (MRI) and radiograph analysis for surgical planning, and meticulous decompression with attention to anatomical landmarks. Additionally, potential complications like dural tears and intra-operative bleeding, as well as their bailout options, are discussed. Revision decompression for degenerative lumbar disease traditionally involves open or microscopic approaches. Endoscopic spine surgery (ESS) offers advantages over these approaches, including improved visualisation, reduced soft tissue disruption, and better short-term outcomes. Overall, RUBELSS presents a viable alternative for revision decompression, offering advantages over traditional techniques with superior short-term outcomes, faster operating times, and reduced complications.

## Introduction

Prevalence of lumbar degenerative disc disease (LDDD) ranges from 2.4% to 5.7% worldwide [1] and has continued to increase in recent years. Diagnosis of LDDD is via plain radiographs or with more sensitive magnetic resonance imaging (MRI) scans showing typical features [2]. LDDD can be asymptomatic but may also present with nonradicular lower back pain, radicular symptoms, and weakness. These symptoms may result from disc herniation [2,3], which, after a trial of conservative management, is often amenable to surgical intervention [4].

Surgical revision rates for patients who have previously undergone open decompression range from 7.4% to 15.1% due to reasons including recurrent herniated disc, progression of end plate spurs, thickening of flaval tissue, scar tissue, worsening spondylolisthesis, conferred instability post-index surgery, adjacent segment disease, and inadequate initial decompression [5,6]. In addition, post-operative outcomes after revision surgery are less favorable with increased pain, disability, or dissatisfaction [5].

Conventionally, revision decompression has been limited to open [5] and microscopic approaches due to familiarity, although there have been recent studies on patients undergoing minimally invasive surgery (MIS) techniques such as endoscopic spine surgery (ESS) for revision surgery [7-10]. Revision surgery can be technically challenging and is associated with more procedure-related complications [11,12]. A recent study by Lee et al. [13] proposed an algorithm for selecting the surgical approach for LDDD revision, concluding that not all types of LDDD are amenable to MIS. As such, the use of MIS for revision LDDD remains controversial due to limited (theoretical) evidence and the relevance of MIS techniques in the revision of different spinal levels [14].

However, ESS has been shown to improve short-term outcomes, including a lower risk of nerve injury, dural tears, and infection, along with a reduced length of hospitalisation [7,15-19]. Dural tears can also be managed endoscopically [20-22]. Nevertheless, ESS has a steep learning curve and is associated with higher complication rates, particularly when performed by less experienced surgeons.

We propose that revision ESS offers specific advantages over conventional approaches, although it presents certain technical challenges. This technical note outlines our approach and technique for revision unilateral biportal endoscopic lumbar spine surgery (RUBELSS), highlighting key procedural insights, operative pearls, and potential pitfalls.

## Technical report

Relevant endoscopic approaches

Depending on the site of compression, various endoscopic approaches may be considered, including transforaminal, interlaminar, or contralateral over-the-top foraminal approach [15,22-24]. These MIS approaches allow for good clinical outcomes with quicker return to work and little to no post-operative complications noted, improved pain and immediate mobilisation [15,23]. The decision on which approach to use is determined by the presence of previous surgery and the site at which the operation was carried out. Careful attention is paid to the extent of previous decompression and the approach used to more accurately localise the new approach. A different endoscopic approach to the index surgery can be considered to circumvent going through scar tissue, which tends to present challenges in localising the anatomical positions, and may result in an inadvertent dural injury.

Pre-operative planning

**Figure 1:**
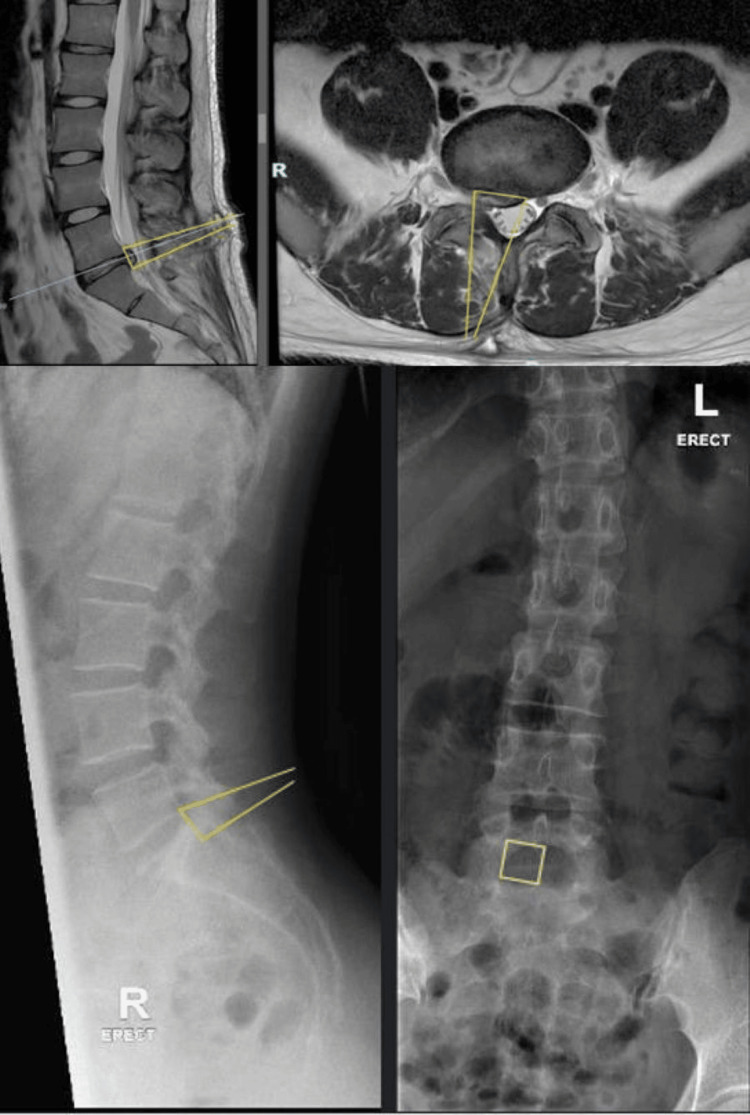
Pre-operative planning showcasing the approach and extent of decompression on MRI prior to a right L5-S1 revision endoscopic decompression and discectomy (top) in sagittal (left) and axial (right) cuts; superimposed on X-ray films (bottom) in lateral (left) and anterior-posterior (right) views MRI: magnetic resonance imaging

Radiographs and MRIs are performed pre-operatively. The MRI is reviewed to identify the cause of the neural compression, e.g., disc, scar tissue, flaval hypertrophy, or endplate spurs. Using VueMotion, the MRI is directly compared to the radiograph, and lines are drawn to see the angle of approach, confirmation of the level and laterality and extent of decompression necessary to achieve symptomatic relief (Figure 1). This allows for simple and easy reference intra-operatively using fluoroscopy.

Anaesthesia and positioning

The patient is put under general anaesthesia. The patient is then turned 180 degrees and positioned prone at 18 degrees flexion on a radiolucent ProAxis spinal table to open the posterior elements, including the interlaminar space and foramen. This is to avoid potential positioning-related complications such as pressure injuries or vision loss. The operating surgeon stood on the pathological side initially.

Approach

Skin incisions are marked 1 cm above and below the disc space for an interlaminar approach and at the foramen level for a transforaminal approach under Image Intensifier (II) guidance. The patient is cleaned and draped, and the skin incision is made according to skin markings. Two portals are established by dissecting the paraspinal muscles with serial dilators until the interlaminar or extra-foraminal space is reached, confirmed by direct visualisation. Position and the approach may be confirmed with intra-operative biplanar fluoroscopy alongside the pre-operative planning. The radiofrequency probe is used to achieve adequate haemostasis, and the borders of the operative field are established [20]. 

Decompression

**Figure 2:**
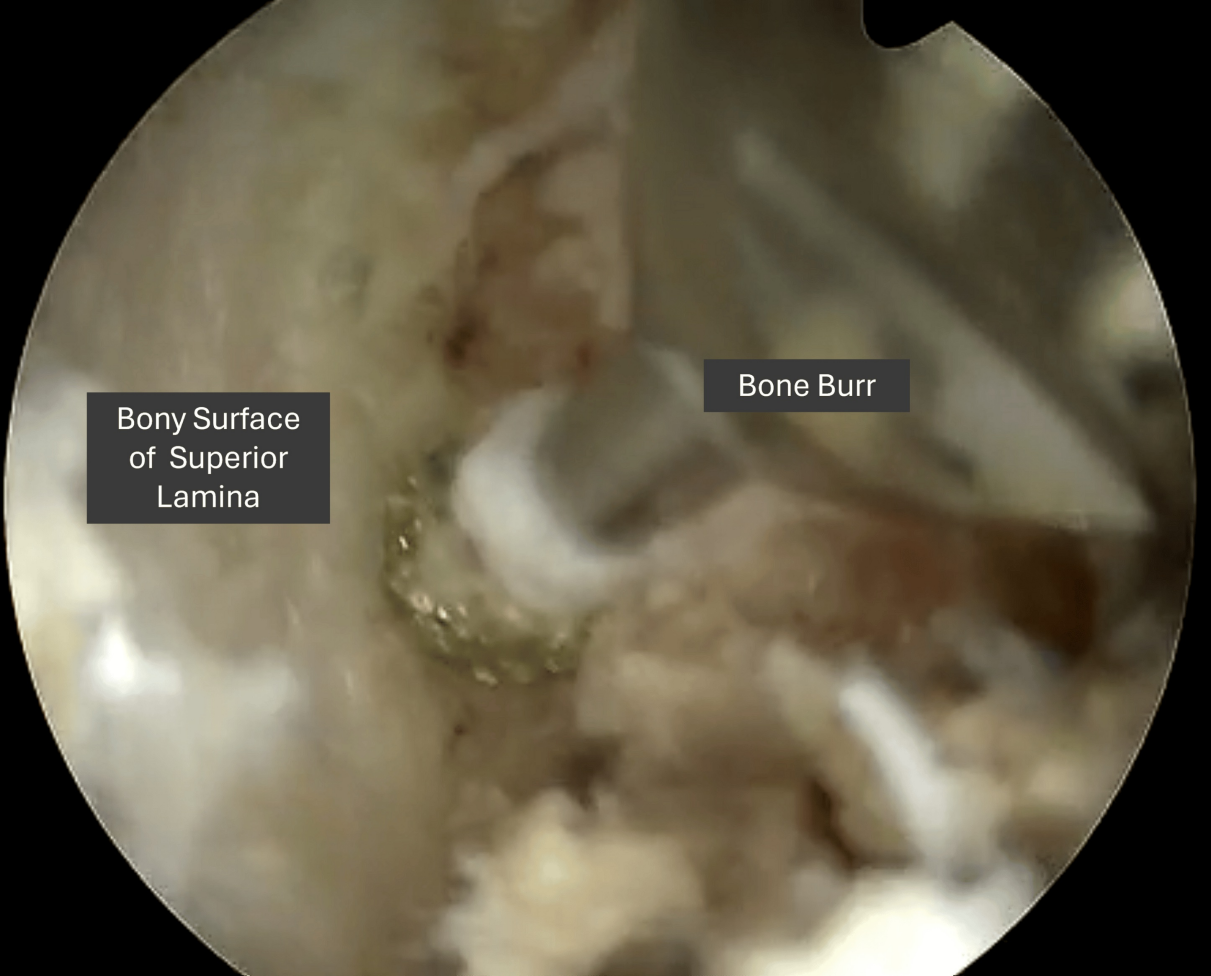
Intra-operative images showing close instrument-to-bone contact to reduce risk of plunging into the spinal canal

A thorough understanding of the bony anatomy is especially critical in RUBELSS, as prior surgical intervention may have altered the native anatomical landmarks. This should be corroborated with pre-operative MRI scans and confirmed with intra-operative fluoroscopy prior to commencing decompression. Once the working window is established, decompression can begin. Similarly to native decompressive endoscopic surgery, a high-speed burr followed by Kerrison rongeurs is helpful for resection of the bones such as the lamina and medial facet joint. Particularly in revision cases where bony landmarks may be altered and the depth of instruments may not be certain, it is recommended to maintain close instrument-to-bone contact (Figure 2) when performing the decompression to reduce the risk of plunging into the spinal canal.

**Figure 3:**
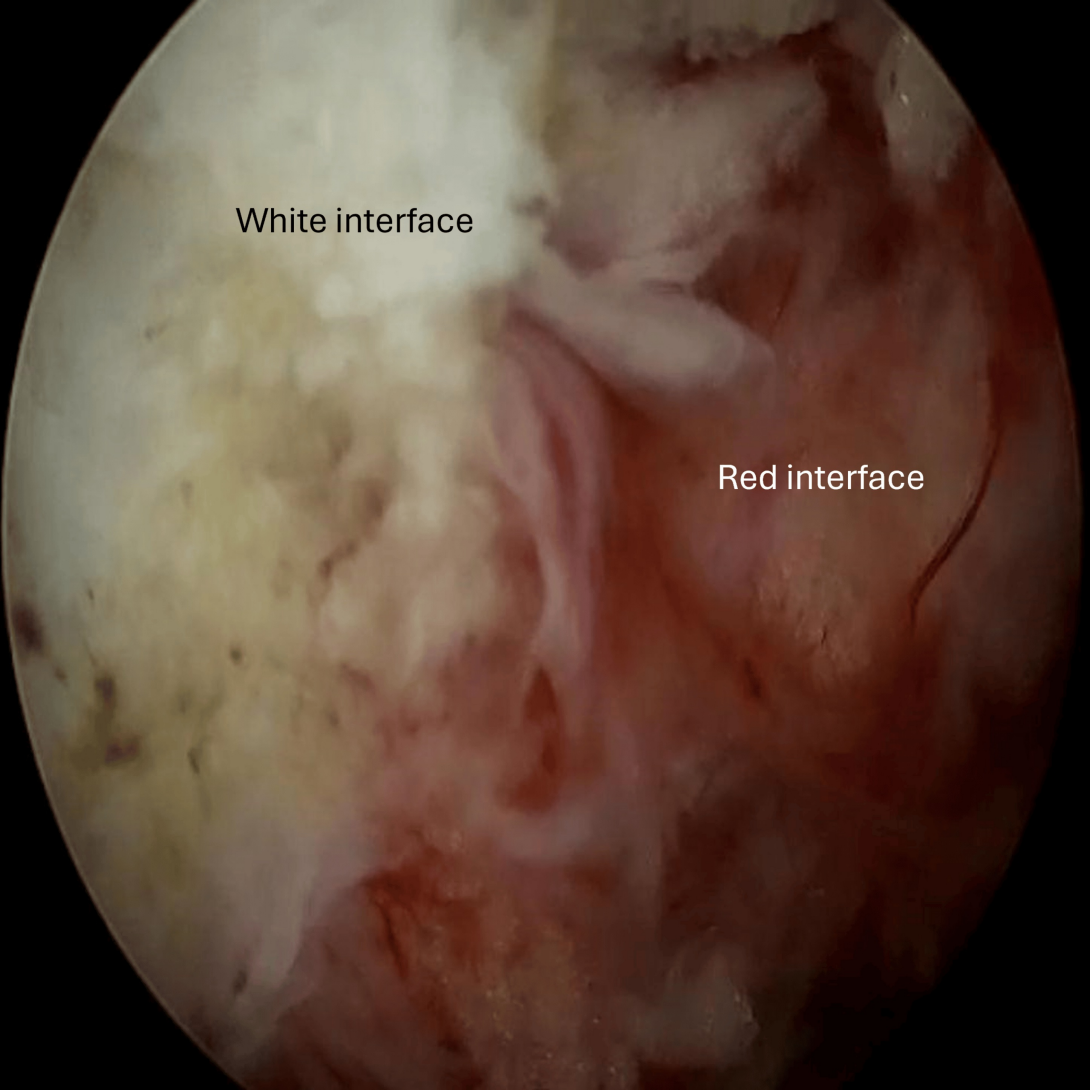
Intra-operative photos showing the establishment of the red white interface

Past the bony tissue window, the soft tissue decompression begins at the depth of the ligamentum flavum. Commonly at this point, previous flaval tissue is now replaced with scar tissue, and it is often difficult to delineate the proximity of the dural sac and spinal canal. The authors find it helpful to establish the red-white interface (Figure 3), which helps to differentiate between scar tissue and critical structures within the spinal canal.

**Figure 4:**
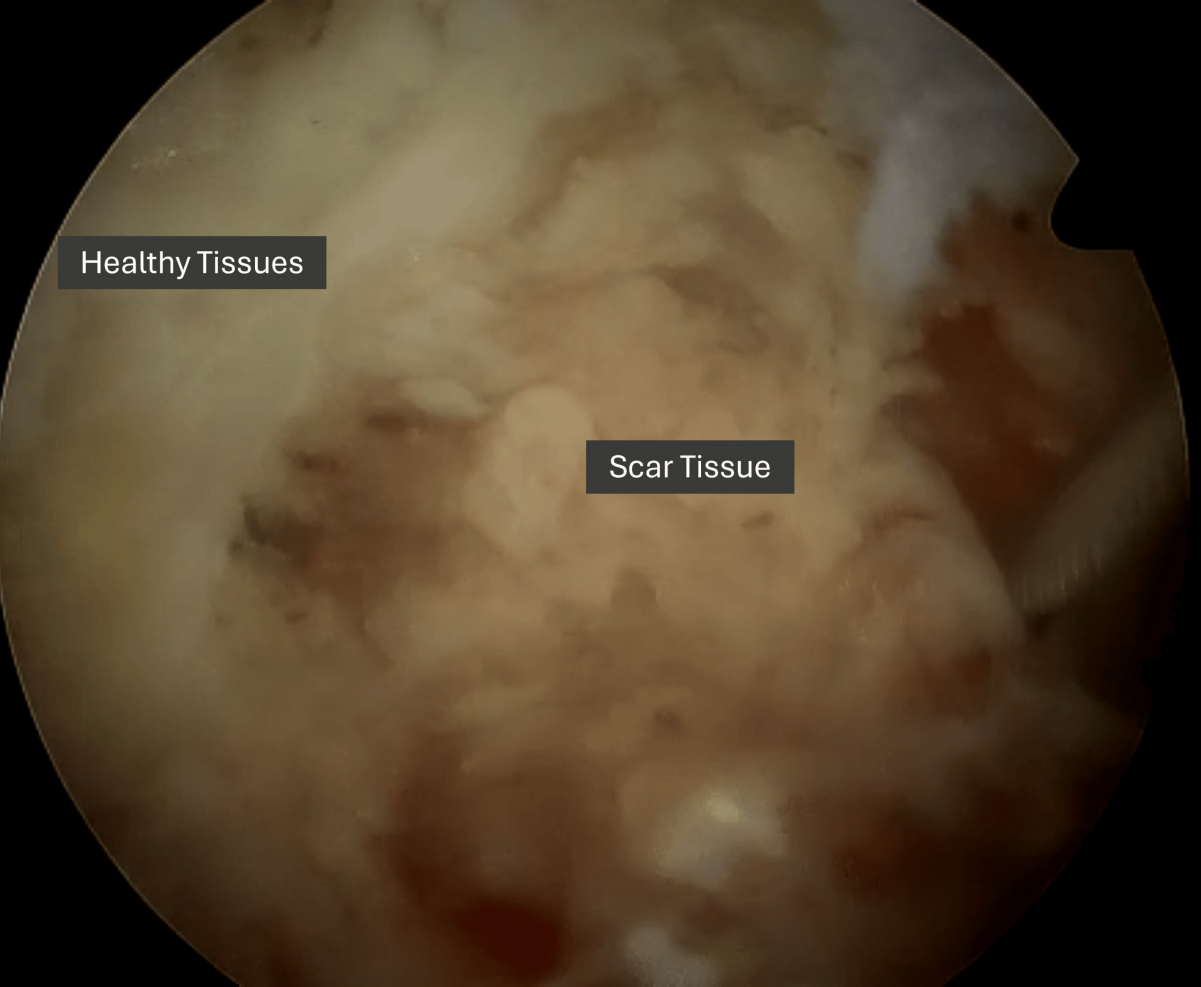
Intra-operative photo showing increased magnification of the operative field by bringing the endoscope closer to allow for separation of tissue planes

A mixture of a curette, Kerrison rongeur, and nerve hook can be helpful to debride this scar tissue carefully to reveal the spinal canal. Bringing the endoscope closer to the operating field allows positive water pressure to separate tissue planes (Figure 4) to better delineate scar and healthy tissue.

**Figure 5:**
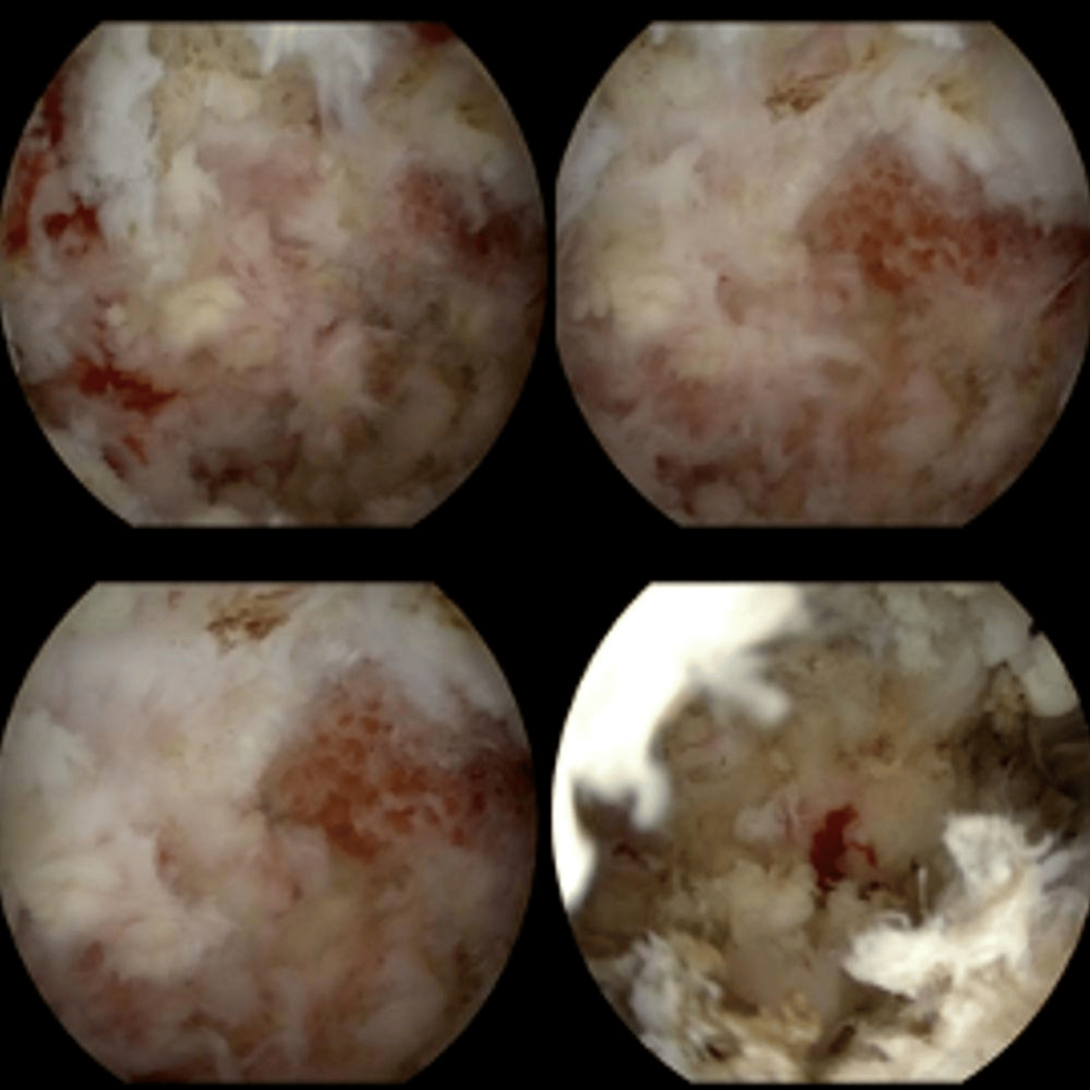
Intra-operative endoscopic images showing the offending recurrent disc being sequentially removed (top left to right, bottom left to right)

Once the dural sac has been successfully decompressed and retracted medially, the underlying disc and associated endplates can be directly visualised and accessed. At this point, if required, a discectomy may be performed. A microcutter or bipolar probe is used to open the annulus or scar tissue, followed by piecemeal removal of the herniated disc and debris (Figure 5).

**Figure 6:**
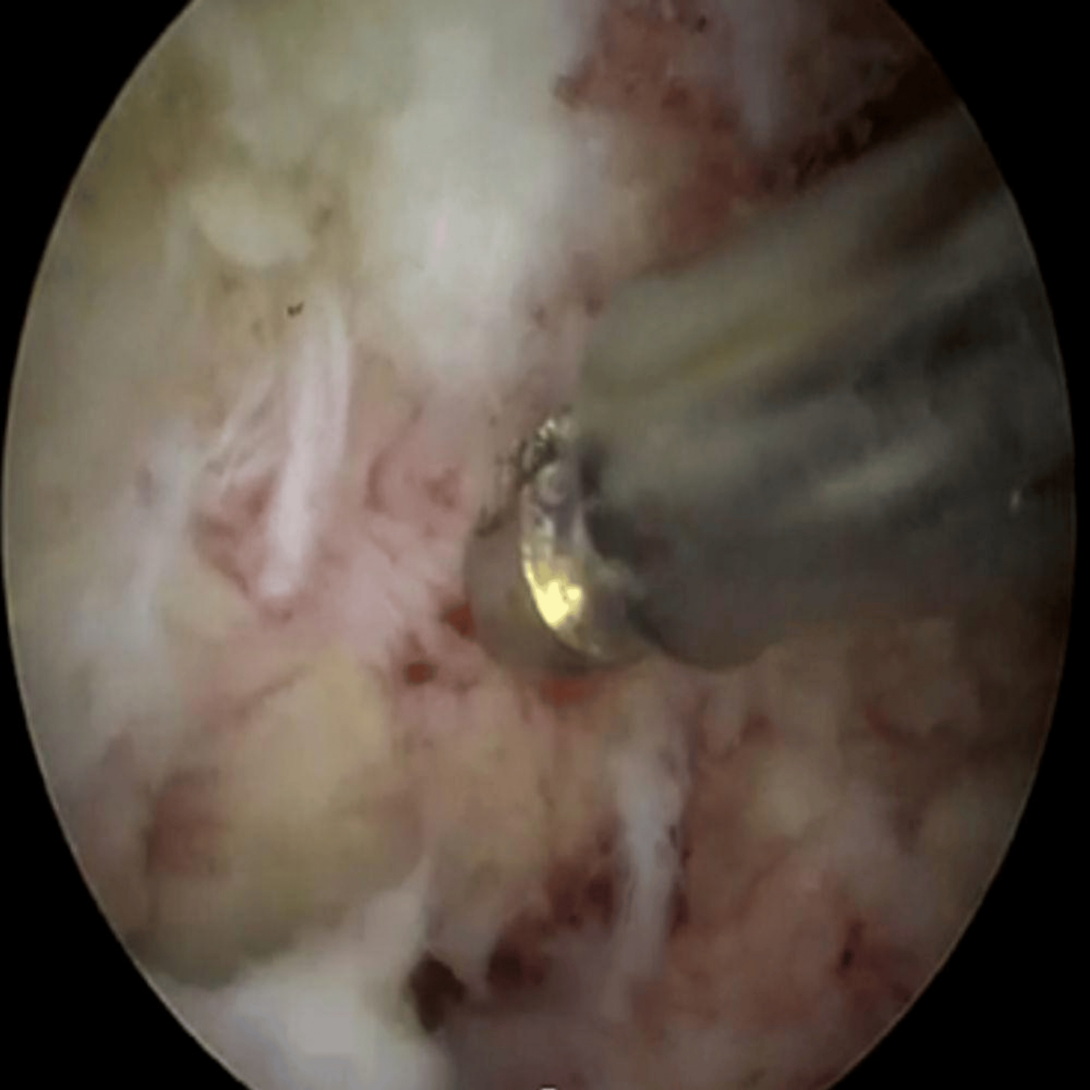
Intra-operative image showing early meticulous haemostasis

**Figure 7:**
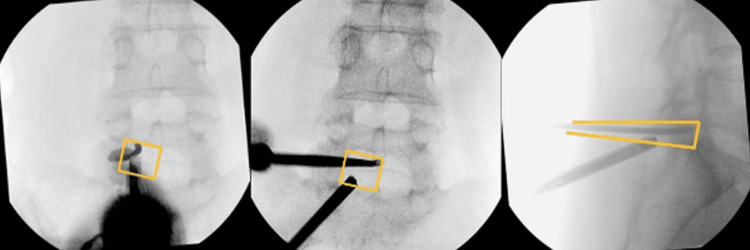
Intra-operative fluoroscopy confirming adequate decompression based on pre-operative planning on AP views (left, middle) and on lateral view (right) AP: anteroposterior

Radiofrequency ablation can be used to perform an annuloplasty, reducing nociceptive input and thus reducing discogenic pain [25]. In addition, adjuncts such as fibrin sealants can reduce the risk of recurrence of prolapsed intervertebral discs [26]. At this point, evaluation of the adjacent endplate spurs should take place, with careful resection if necessary. It is paramount to have early meticulous haemostasis (Figure 6), which can be achieved with radiofrequency ablation and bone wax to ensure clear visibility throughout the procedure [20]. Following this, the extent of decompression is confirmed with intra-operative fluoroscopy (Figure 7) and compared with the superimposed pre-operative images. Additionally, the nerve roots and dural sac are both mobilised and confirmed to be decompressed. The endoscope and devices are withdrawn, and the skin is closed with sutures of the surgeon’s preference.

Dealing with complications and bailout options

**Figure 8:**
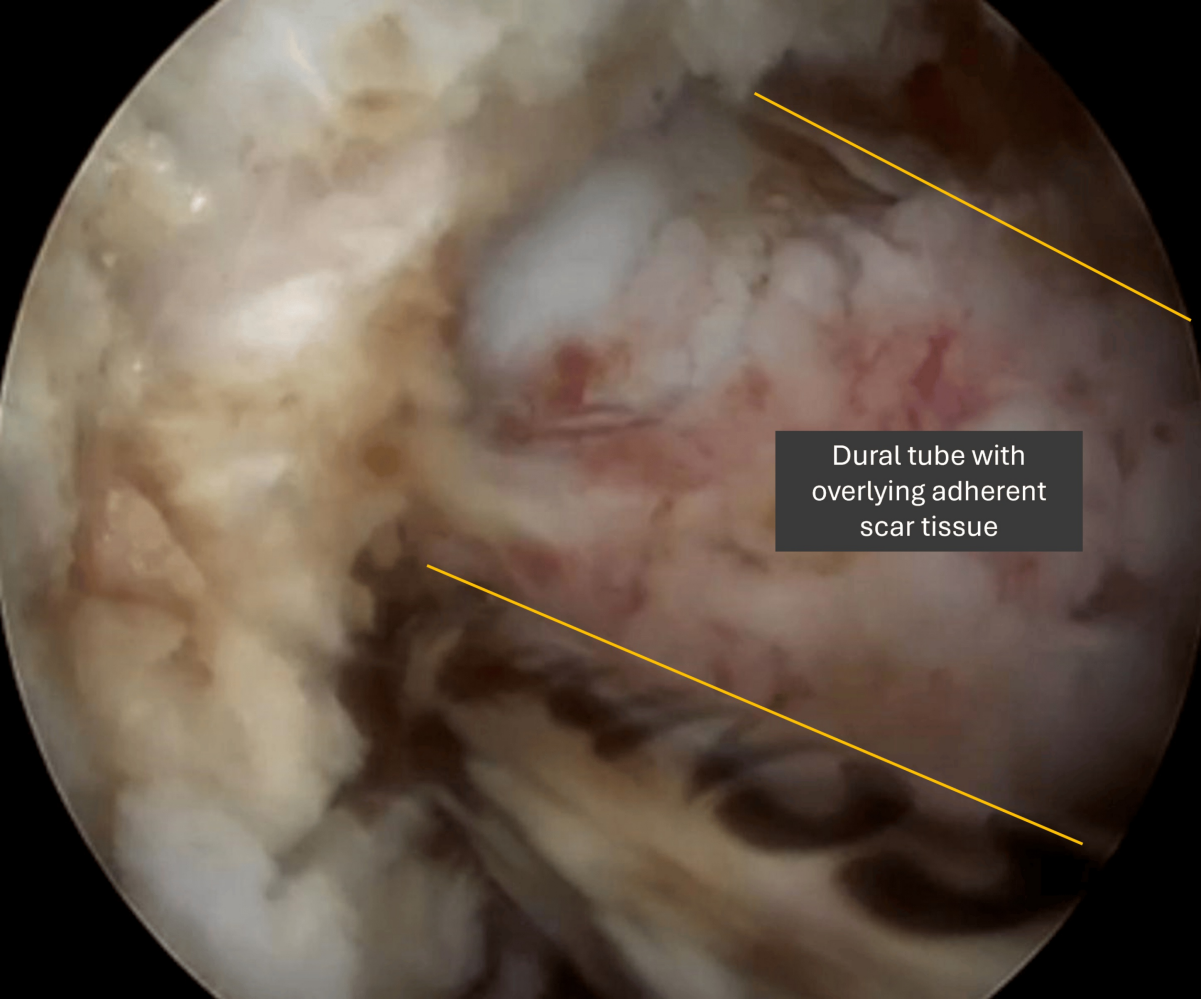
Intra-operative image showing thin layer of scar tissue left behind to allow for combined free floating

**Figure 9:**
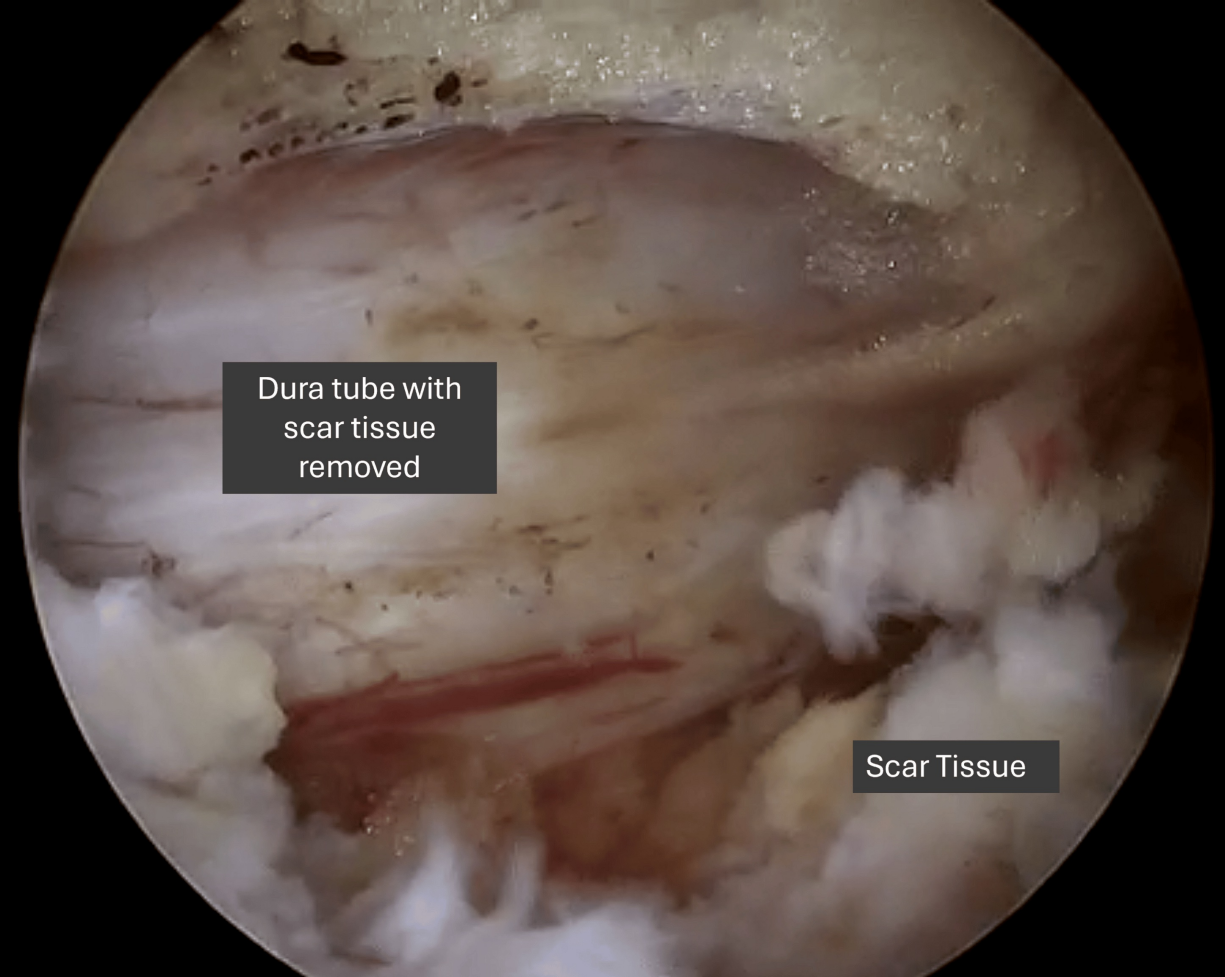
Intra-operative image showing complete removal of the adherent scar tissue from the dural sac

To minimise the risk of dural tears, a thin layer of scar tissue adherent to the dura may be intentionally preserved, allowing the dura and scar to move together as a single, free-floating unit (Figures 8-9). Reducing the speed of manoeuvring during the dissection process under direct visualisation can also mitigate the risks of dural injury. However, in the case of a dural tear, it is helpful to have human thrombin and fibrinogen sponge sealant on standby to act as a patch repair. Intra-operative bleeding may be particularly difficult to overcome, and it is helpful to have fibrin on standby to achieve haemostasis in the event that the area of bleeding is difficult to localise [20].

## Discussion

LDDD is commonly managed using a variety of decompressive techniques, with or without instrumentation or fusion, and these procedures may be performed through open, microscopic, or MIS approaches [27]. To the best of our knowledge, there has been no set criterion for the treatment of LDDD by RUBELSS. A recent study by Yingsakmongkol et al. [28] describes the criteria for surgical intervention via LLIF decompression in symptomatic individuals, which include pain relief of 50% or more in the supine position, no significant weakness, and disc discrepancy >1 mm between the supine and upright positions. However, there has also been no conclusive evidence for patient-specific characteristics which may influence pre-operative states and outcomes after treatment [28]. More research involving randomised controlled trials comparing RUBELSS to open revision decompression has to be done to look into the various factors that may influence the need for surgical treatment.

Studies show that RUBELSS has several advantages over microsurgical techniques [16,17,29]. Ruetten et al. compared the revision full endoscopic (FE) and revision microsurgical technique (MI). The study showed no difference in clinical outcomes with shorter operation times (24 min FE, 58 min MI), decreased postoperative pain, pain medication, complications (6% FE, 21% MI), and work disability (28 days FE, 52 days MI) with no significant difference in recurrence [16]. Similarly, Lee et al. compared endoscopic lumbar discectomy and open lumbar microdiscectomy, showing significantly shorter mean operating times, hospital stay, and complication rates [17].

RUBELSS also enables an alternative approach to the index surgery by circumventing previously operated (scarred) tissue, owing to its enhanced manoeuvrability and lower incidence of dural tears [30]. In contrast, non-MIS techniques face the issue of differentiating scar tissue from normal tissue with a higher risk of dural tears and larger dissection of posterior structures to confirm bony landmarks. This can lead to iatrogenic instability requiring fusion surgery [31]. ESS, with its increased manoeuvrability and visualisation, allows for selection of alternative approaches and can minimise the need to strip away large portions of tissue, allowing for focal decompression without compromising stability [15,32]. Its increased magnification and positive water pressure also allow for better delineation of scar tissue by separating tissue planes. 

Some drawbacks of revision endoscopic surgery include greater total in-hospital costs when compared to open surgery [33] and a steep learning curve with a higher complication rate in less experienced users [20]. Table 1 summarises the pearls and pitfalls of RUBELLS. 

**Table 1:** Summary of the pearls and pitfalls of RUBELSS RUBELSS: revision unilateral biportal endoscopic lumbar spine surgery

	Pearls	Pitfalls
Pre-op	Marking the extent of decompression on MRI and radiograph allows the surgeon to easily confirm the angle of approach, level, laterality, and extent of decompression required intra-operatively	-
	Consider alternative approaches to go through untouched virgin tissue rather than scar tissue if the pathology allows	
Post-op	Intra-operative fluoroscopy with reference to pre-operative planning should always be used when there is any uncertainty	Poor haemostasis can lead to significant difficulties and lengthen operative timing, and use of radiofrequency ablation, bone wax, coagulation gels, or adjuncts can be helpful
	Establishment of the white (scar tissue) and red (healthy dural sac/virgin tissue) is crucial to guide decompression	
	When encountering difficulties in visualizing tissue planes between scar tissue and healthy tissue, bringing the endoscope closer to the operating field can allow positive water pressure to separate tissue planes	
	Consider fibrin sealant and radiofrequency ablation for annuloplasty after discectomy to reduce the risk of recurrence	
Dealing with complications/bailout options	If scar or flavum tissue adheres too tightly to the dura sac, a thin layer of scar tissue adherent to the dural sac can be left so as to not risk dural tears	-
	In the case of a dural tear, it is helpful to have human thrombin and fibrinogen sponge sealant should be on standby which can act as a patch repair	

## Conclusions

ESS provides surgeons with a minimally invasive approach, high magnification, improved visibility, positive water pressure, and manoeuvrability, proving highly useful in revision decompression for degenerative lumbar disease compared to traditional open or microscopic surgery. However, the present study’s findings are procedural and illustrative only, and not outcome-based. Yet, leveraging these advantages, ESS potentially offers superior short-term outcomes, faster operative times, and reduced complications, as corroborated in current literature. Careful pre-operative planning, sequential decompression, and mindfulness of complications and bailout techniques are paramount when electing to utilise RUBELSS.
